# Nonmuscle Tissues Contribution to Cancer Cachexia

**DOI:** 10.1155/2015/182872

**Published:** 2015-10-07

**Authors:** Josep M. Argilés, Britta Stemmler, Francisco J. López-Soriano, Silvia Busquets

**Affiliations:** ^1^Cancer Research Group, Departament de Bioquímica i Biologia Molecular, Facultat de Biologia, Universitat de Barcelona, 08028 Barcelona, Spain; ^2^Institut de Biomedicina de la Universitat de Barcelona, Barcelona, Spain; ^3^BSA Nutrition Centre, 08195 Barcelona, Spain

## Abstract

Cachexia is a syndrome associated with cancer, characterized by body weight loss, muscle and adipose tissue wasting, and inflammation, being often associated with anorexia. In spite of the fact that muscle tissue represents more than 40% of body weight and seems to be the main tissue involved in the wasting that occurs during cachexia, recent developments suggest that tissues/organs such as adipose (both brown and white), brain, liver, gut, and heart are directly involved in the cachectic process and may be responsible for muscle wasting. This suggests that cachexia is indeed a multiorgan syndrome. Bearing all this in mind, the aim of the present review is to examine the impact of nonmuscle tissues in cancer cachexia.

## 1. Cachexia as an Energy-Wasting Syndrome

Cachexia, from the Greek: “*kakos*” and “*hexis*,” meaning “bad condition,” is a multiorgan syndrome associated with cancer and other systemic diseases such as sepsis and renal failure and characterized by at least 5% body weight loss due to muscle and adipose tissue wasting and inflammation [1]. Abnormalities associated with cachexia include alterations in carbohydrate, lipid, and protein metabolism [2]. Cancer cachexia has been characterized as a syndrome associated with loss of muscle with or without loss of fat mass. Other disorders associated with cachexia are anorexia, inflammation, insulin resistance, and increased muscle protein [2]. Another defining characteristic is that cachexia cannot be fully reversed by conventional nutritional support and leads to progressive functional impairment [3]. Thus, it can be concluded that cachexia is caused by an energy imbalance which is the result of both decreased food intake, due to marked anorexia, and increased energy expenditure caused by a highly hypermetabolic state. Blum et al. [4] in a recent meta-analysis of cancer cachexia conclude that current data support a modular concept of cancer cachexia with a variable combination of reduced nutritional intake and catabolic/hypermetabolic changes.

Cachexia occurs in the majority of terminal cancer patients and is responsible for the deaths of 22% of cancer patients [5]. Importantly, survival of cancer patient suffering from different types of neoplasias is dependent on the amount of weight loss [6]. Therefore, cachexia represents an important factor in the treatment of a cancer patient, affecting not only survival, but also the efficacy of anticancer treatment, quality of life, and medical costs. Thus there is a strong pressure to better understand the mechanisms that drive cachexia in order to offer cancer patients more effective care.

**Figure 1:**
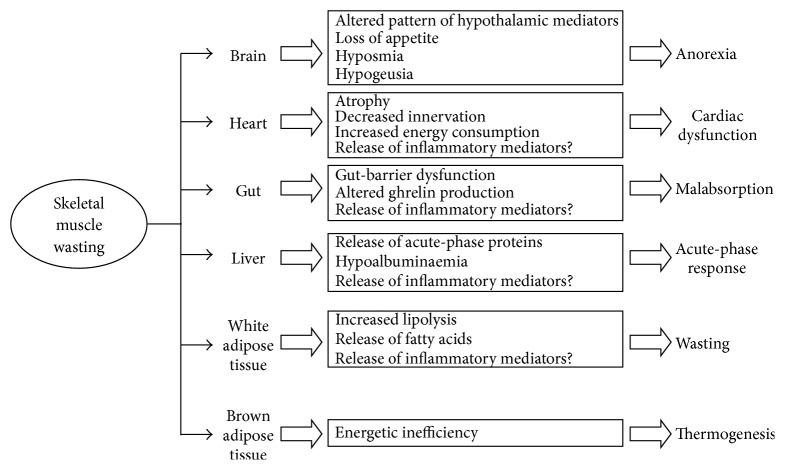
Interactions between different tissues/organs and skeletal muscle in the development of wasting associated with cancer cachexia. The events and metabolic alterations that take place in different tissues/organs during cancer cachexia may be related with the loss of muscle tissue. Indeed, muscle wasting may be influenced by the liver, inflammatory response, and by adipose tissues, particularly white fat. Brown adipose tissue could partially account for the energy inefficiency associated with hypermetabolism in the cancer patient. Brain, basically by modulation of appetite, also contributes to muscle wasting. The gut may be responsible for both malabsorption and changes in ghrelin production and, finally, the heart could also be a source of inflammatory mediators contributing to further muscle wasting.

Here we will discuss the impact of nonmuscle tissues, only the ones that have a certain role; that is, kidney and lung do not seem to have a role in cancer cachexia. Indeed recent developments suggest that tissues/organs such as adipose (both brown and white), brain, liver, gut, and heart are directly involved in the cachectic process and may be responsible for muscle wasting. This suggests that cachexia is indeed a multiorgan syndrome (Figure 1).

## 2. Brain

Although a recent study involving 1853 cancer patients [7] did not find common genetic causes in appetite loss in cancer patients, cytokines, neuroendocrine changes, and tumour mediators are the main signals involved in appetite depression in cachexia. Additional factors contributing to the anorectic state are altered taste perception, therapy-induced side effects [8], depressed motor activity, possible mechanical interference on the gastrointestinal tract, and, of course, psychological factors [9]. Indeed, patients with cachexia often experience psychological distress as a result of the uncertainties of the disease, its diagnosis, its treatment, and its anticipated and final outcome [9]. This psychological state, which often involves depression, is bound to affect food intake. Both the limbic system and the brain stem participate in the regulation of appetite and energy balance. Thus, morphologically defined regions of the hypothalamus, the arcuate nucleus (ARC), the paraventricular nucleus (PVN), the dorsomedial nucleus (DMH), the ventromedial nucleus (VMH), the lateral hypothalamic area (LHA), and the perifornical area (PFA), appear to play a major role in the regulation of body weight. There are two primary neuron types within the ARC that integrate signals of nutritional status and influence energy homeostasis: a subpopulation of neurons in the medial ARC expresses the orexigenic neuropeptides (neuropeptide Y (NPY) and agouti-related peptide (AgRP)). More laterally there is a second subpopulation that inhibits food intake via the expression of cocaine- and amphetamine-regulated transcript (CART) and proopiomelanocortin (POMC), which is processed to melanocyte stimulating hormone (MSH). Specific neuropeptides are involved in the signalling of the neuronal circuits within these regions of the hypothalamus, for instance, corticotrophin-releasing hormone (CRH), thyrotropin-releasing hormone (TRH), NPY, brain-derived neurotrophic factor (BDNF), orexin, and melanin-concentrating hormone (MCH). The brain stem also plays an important role in the regulation of energy balance. Reciprocal connections are present, in an extensive way, between the hypothalamus and brain stem, particularly at the level of the* nucleus tractus solitarii* (NTS). This nucleus is in close anatomical proximity to the area postrema, a circumventricular organ that has an incomplete blood brain barrier. Like the ARC, the NTS is therefore located in an ideal place to respond to peripheral circulating signals but in addition also receives vagal afferent signals from the gastrointestinal tract and afferents from the glossopharyngeal nerves.

Therefore, brain mediators involved in the control of food intake, appetite, satiation, taste, and smell of food, are responsible for the anorexia of the cancer patient, making the brain a main organ responsible for one of the components of the altered energy balance in cancer patients [10] (Figure 1). Although anorexia represents a very important factor in the development of cachexia, it has to be pointed out that in many cases the use of total parenteral nutrition does not stop the loss of body weight [11]. In addition to anorexia, the hypothalamus via the melanocortin system may contribute to muscle wasting via neuronal output, as suggested by different animal studies [12, 13].

## 3. Gut

Gut-barrier dysfunction is a syndrome characterized by both breakdown and leakage of the gut epithelial barrier, leading to systemic inflammation due to the entry of bacterial cell wall components (endotoxin or lipopolysaccharide), or intact bacteria into the circulation. Gut-barrier dysfunction is often observed during the course of cancer cachexia [14] (Figure 1) and is partially connected to radiochemotherapy treatment. Additionally, tumour growth or macrophage infiltration at the level of the intestinal wall may affect gastrointestinal permeability, either locally or throughout the intestine via alterations in epithelial tight junctions [15]. From this point of view, tight junction proteins, such as ZO-1 and occludin, show decreased expression in tumour-rich regions of the intestine and colon in humans [16]. Decreases in tight junction proteins would increase permeability and allow passage of large molecules such as lipopolysaccharide (LPS) into the lymphatic circulation. Changes in mucin secretion and profiles in gastrointestinal carcinomas may become a source of inflammation in the course of cancer cachexia [17]. Indeed, gut-barrier dysfunction may lead to endotoxemia and, therefore, increased inflammation in cancer patients [14].

In addition to the gut-barrier dysfunction syndrome, recent studies support a role for gut microbiota in cancer cachexia. Indeed, decreased levels of bacteria, which have immunomodulating properties, are decreased during experimental cancer cachexia [18]. The existence of a gut-microbiota-skeletal muscle axis has been reported [19]; in fact, gut microbiota generates metabolites that can reach skeletal muscle and influence energy expenditure in the muscle cells [20].

Other aspects related with the gut may be beneficial for cachectic patients. Ghrelin is the first identified circulating hunger hormone that influences body weight regulation via a vagal pathway [21]; it is a gastric hormone initially identified in the rat stomach, in 1999, as an endogenous ligand for the growth hormone secretagogue receptor (GHSR) [21]. Under fasting conditions, the stomach, through endocrine cells located in the* antrum*, secretes ghrelin into the bloodstream. The hormone acts as a “hunger” mediator that signals the gastrointestinal fuel status from the periphery to the central nervous system in order to stimulate food intake and to adjust energy balance through decreasing energy expenditure [22]. Ghrelin also binds to the GHSR1a splice-variant that is enriched in the hypothalamus, as well as other brain regions. In the hypothalamus, at the level of the ARC, ghrelin contributes to enhanced food intake by activating orexigenic mediators such as NPY, gamma-aminoisobutyrate (GABA), and AgRP, and inhibiting anorexigenic mediators such as POMC, MSH, and CART [23]. The GHSR1a receptor is also present on vagal afferents [24] and, therefore, there is also strong evidence that ghrelin has a peripheral effect on satiety by affecting the mechanosensitivity of upper gastrointestinal vagal afferents, making them less sensitive to distension which can result in overeating. Ghrelin also has potent effects on fat storage. Ghrelin activates white adipocytes [25] while doing the opposite to brown adipocytes, therefore contributing to decreased energy expenditure [26]. These effects are related with the capacity of the hormone to stimulate growth hormone (GH) release from the anterior pituitary [27]. In addition, ghrelin increases insulin-like growth factor 1 (IGF-1) by stimulating its own receptor. These two factors are major signals being related with the regulation of energy homeostasis. The effects of ghrelin on GH and IGF-1 may be linked to the capacity of the hormone to prevent increases in protein degradation, through different components of the proteasome such as MuRF1 and MAFbx, at the level of skeletal muscle. This is particularly relevant to cancer cachexia since muscle wasting occurs mainly through activation of the ubiquitin-dependent proteolytic system. Ghrelin levels are elevated in cancer cachectic patients with neuroendocrine [28], gastric [29, 30] and lung [31] tumours. These elevated levels could represent a counterregulatory mechanism to fight anorexia associated with tumour growth. It is, in fact, an endocrine response to the so-called “ghrelin resistance” found in cancer patients. Indeed, this is, in part, the reason for the high doses of ghrelin used in clinical studies to counteract anorexia in cancer. In clinical practice the use of ghrelin and ghrelin agonists has led to promising results in cancer cachectic patients. Ghrelin treatment improves physical performance and muscle force indicating that the peptide is the best candidate for muscle wasting treatment either alone or in combination with other drugs or nutritional strategies [32–37]. Therefore, future research is needed to search for the optimal combination. Cancer cachexia is a multiorgan syndrome affecting not only skeletal muscle but also adipose tissues, heart, intestine, kidney, and liver. In fact, the final cause of death in cachectic cancer patients is, apart from the primary tumour itself, either sudden death (heart arrhythmias, hypoventilation), thromboembolic events (platelet aggregation), cardiorenal alterations (kidney dysfunction), or compromised immune function (immunosuppression). Ghrelin has a beneficial effect in all of the referred tissues. The only concern in treating cachectic cancer patients relates to the fact that ghrelin may contribute to tumour cell proliferation [38]. Indeed, ghrelin may increase the levels of growth factors, such as GH and IGF-1 that stimulate tumour growth. Additionally, ghrelin itself may have mitogenic potential. As far as we know, no* in vivo* data has examined the differences in tumour growth following ghrelin or GHS treatment. Long-term, large-scale clinical trials are required to determine whether ghrelin treatment promotes tumour growth.

In addition to the role of the gut ghrelin in the control of food intake, other mediators are bound to be involved in the anorexia associated with the cachexia syndrome. From this point of view, melanocortin-4 receptor [39] or prostaglandins [40, 41] may work independently or alongside ghrelin.

## 4. Liver

The liver plays a key role in regulating whole-body metabolism. Claude Bernard introduced the idea that the liver is the “glucostat” of the organism, by regulating glucose production and levels in mammals. Indeed, the liver can be regarded as the central node of supply and utilization of fuel by the tissues, the direction and flux of which are mediated by the endocrine system [42].

**Figure 2:**
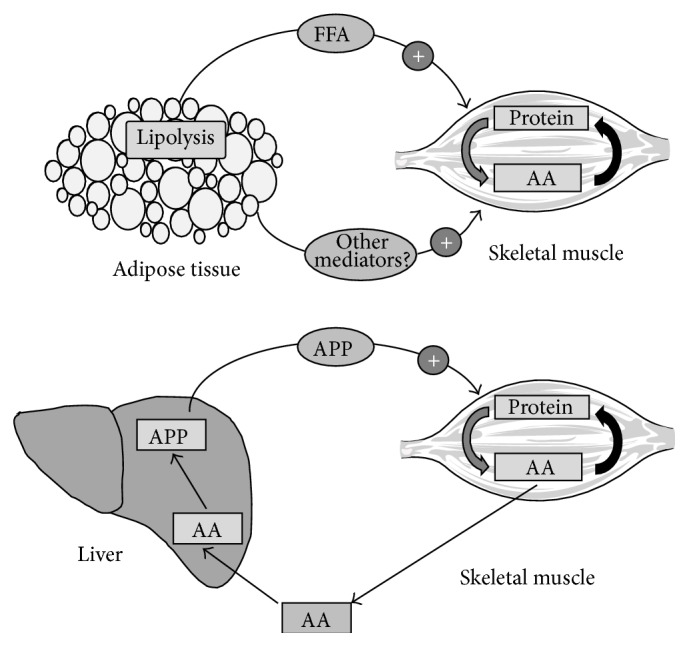
Examples of cross talk between adipose tissue/liver and skeletal muscle during cancer cachexia. Adipose tissue releases some factor(s), possibly fatty acids, that seem to be essential to activate muscle proteolysis. Indeed, recent evidences, using knockout deficient mice, suggest that blocking lipolysis in white fat results in an amelioration of muscle wasting. Similarly, liver acute-phase proteins (APP), such as serum amyloid A (SAA), could participate, alone or in synergy with cytokines, in activating muscle wasting by enhancing protein degradation.

During catabolic conditions, opposing patterns of protein metabolism are observed between skeletal muscle and liver. While skeletal muscle is under negative nitrogen balance, mainly due to enhanced protein degradation, the liver exhibits important changes in the patterns of protein synthesis such as increased production of acute-phase proteins [42]. The enhanced muscle proteolysis drives a large release of amino acids from skeletal muscle, such as alanine and glutamine [43]. While glutamine is taken up by tumour cells to sustain both the energy and nitrogen demands of the growing mass, alanine is mainly channeled to the liver for both gluconeogenesis and protein synthesis [43]. Indeed, liver fractions from tumour-bearing animals show increased production of acute-phase proteins including C-reactive protein (CRP), serum amyloid A (SAA), *α*1-antitrypsin, fibrinogen, and complement factors B and C3 and a decrease in the synthesis of transferrin and albumin, leading to hypoalbuminemia [44]. An acute-phase response is also observed in cancer patients. In addition, in cancer patients with advanced cancer and during the fasting state, the total albumin synthesis rate is unchanged, compared with controls, despite much lower albumin concentrations [45]. Although the function of these proteins is far from being clear, it is known that CRP contributes to the activation of complement factors, enhancement of phagocytosis, and regulation of cell immunity; *α*1-acid glycoprotein inhibits platelet aggregation and phagocytosis and may be involved in spacing collagen fibres; haptoglobin binds to and clears haemoglobin from plasma; *α*1-antitrypsin and *α*2-macroglobulin regulate serine-proteases; and ceruloplasmin is probably involved in copper transport. Recently, a link between SAA, in synergy with interleukin-6 (IL-6), and activation of muscle proteolysis has been described [46] (Figure 2).

During cancer, the patient's inflammatory response (Figure 1) is linked to weight loss and poor performance status. Indeed, many inflammatory mediators are able to influence different metabolic pathways related to cachexia [2, 43]. The liver is an important contributor to the inflammation observed in cancer. Indeed, CRP seems to be a very important prognostic parameter [47–49].

The liver can also contribute to energy inefficiency. Indeed, Dumas et al. found that the efficiency of oxidative phosphorylation in liver mitochondria was decreased in a rat model of peritoneal carcinosis, suggesting that this may also contribute to hypermetabolism, elevated energy expenditure, in cancer-bearing states [50]. These alterations were associated with the content and fatty acid composition of cardiolipins [51]. Indeed, the phospholipid composition and especially cardiolipins are crucial for the mitochondrial energy metabolism. Indeed, cardiolipin is known to provide essential structural and functional support to several proteins involved in oxidative phosphorylation.

Moreover, it has been described that a higher number of CD68 immunoreactive macrophages have been found in liver cross sections of patients with pancreatic cancer and cachexia, suggesting that a crucial interaction between the tumor, peripheral blood mononuclear cells (PBMCs), and the liver, may play a central role in the development and regulation of cachexia [52].

## 5. Heart

Cancer is associated with severe heart alterations. Indeed, tumours implanted in experimental animals result in a decrease of the heart weight [53, 54], accompanied by functional cardiac changes, similar to those found in congestive heart failure. According to Schünemann et al. “cancer fatigue syndrome reflects clinically non-overt heart failure,” clearly attributing a main role for heart abnormalities in the fatigue of cancer patient [55]. Tian et al. suggested that cardiac alterations in a mouse cancer cachexia model include marked fibrosis, disrupted myocardial ultrastructure, and altered composition of contractile proteins, such as troponin I and Myosin Heavy Chain-*α* (MHC-*α*) [56]. Similarly, Mühlfeld et al. using the well-established cachectic tumour rodent model Lewis lung carcinoma observed changes in heart innervation with the total number of axons in the left ventricle being reduced as a consequence of tumour burden [57]. This altered innervation was associated with a reduced expression of nerve growth factor [57]. The impairment of heart function observed in tumour-bearing animals seems to be specifically related to cardiac remodelling. Indeed, Tian et al. using the mouse C26 tumour model showed increased cardiac B-type natriuretic peptide (BNP) and c-fos expression together with decreased Peroxisome Proliferator Activator Receptor-*α* (PPAR-*α*) and its responsive gene Carnitine Palmitoyl Acyl Transferase-1*β* (CPT-1*β*) and a switch from “adult” isoforms (MHC-*α*, GLUT4) to “foetal” isoforms (MHC-*β*, GLUT1) [58]. The heart atrophy seems to be to some extent related with increased cardiac muscle proteolysis, since protein ubiquitination and expression of MuRF-1 and atrogin-1 are elevated [58]. However, Cosper and Leinwand suggest that the cardiac proteolysis is rather caused by increased autophagy [59], in a converse manner to what happens in skeletal muscle. Interestingly, inhibition of NF-*κ*B protects against tumour-induced cardiac atrophy, at least in experimental animals [60]. Cardiac atrophy in experimental cancer cachexia has recently been related with a high affinity activin type 2 receptor (ActRII) that mediates the signalling by a subset of Transforming Growth Factor-Beta (TGF-*β*) family ligands including myostatin, activin, and GDF11. Blocking pharmacologically this receptor reverses cancer-induced atrophy of the heart [61].

In addition to cardiac atrophy, Drott and Lundholm observed an increase in oxygen consumption, most likely related with the anaemia that very often is present in cancer patients, in the heart of an experimental cancer rodent model [62]. Important ultrastructural changes were also observed, such as an increase in the ratio of myofibrils/mitochondria and sarcomeric alterations, similar to those observed during cardiac failure. The increased oxygen consumption can to some extent be associated with increased energy expenditure, thus making the heart an additional organ involved in generating energy inefficiently (Figure 1). Indeed, heart rate seems to be elevated in cancer patients [63]. In fact, this parameter seems to be a very effective measure of cancer death risk. The mechanisms that could explain the association between heart rate and cancer mortality are unclear. Heart rate increase might be a marker of chronic stress and anxiety, which represent a natural consequence of the disease.

## 6. Adipose Tissues

### 6.1. White Adipose Tissue (WAT) and Muscle Wasting

Previous studies have emphasized the cross talk between adipose tissue and skeletal muscle. Indeed, signals released from both tissues, that is, Tumour Necrosis Factor-*α* (TNF-*α*), IL-6, and interleukin-15 (IL-15), may participate in a reciprocal manner in the regulation of adipose and muscle tissue mass [64, 65]. Irisin, a protein produced both in skeletal muscle and adipose tissue in response to exercise, is able to stimulate browning of adipose tissue [66]. The release of irisin seems to be promoted by the transcriptional coactivator PPAR-*α* coactivator-1 alpha (PGC-1*α*) [67]. Very interestingly, the blockade of myostatin drives browning of adipose tissue through activation of the PGC-1*α*-irisin pathway [68]. Alterations in the balance of the signals could well be associated possibly with obesity, diabetes, or cachexia [64, 65]. Indeed, the adipocyte releases TNF-*α* and other cytokines that have a direct effect on muscle metabolism. Similarly, skeletal muscle releases IL-6, IL-15, and other signals that interfere with fat metabolism [64, 65]. Loss of fat mass is a key feature of cancer cachexia and the mechanism that drives this is multifactorial. On the one hand, lipolysis is activated in the adipocyte, which reduces its cellular volume [69]. Lipolysis may be favoured by a dramatic decrease in perilipin [70], a protein that acts as a protective coating from different lipases. The intense lipolysis is accompanied by changes in the expression of genes that regulate energy turnover, cytoskeleton, and extracellular matrix, suggesting high tissue remodelling. Altogether, this results in not only a net loss of the triglyceride depot, but also a change in the phenotype of the fat cell. On the other hand, fat depletion associated with cancer is linked with a decrease uptake of VLDL and chylomicron triacylglycerol due to a decrease in lipoprotein lipase (LPL) activity [2]. The increased fat removal is accompanied by a decrease in the rate of* de novo* lipogenesis in the adipocyte [2]. Interestingly, during cachexia a concomitant inhibition of adipogenesis takes place, possibly triggered by PPAR-*α* [71].

Both hormonal changes, insulin resistance and hyperglucagonemia, and release of proinflammatory cytokines seem to be responsible for the changes in adipocyte metabolism. In addition, both in experimental animals and humans, a zinc-*α*2 glycoprotein (ZAG) has been associated with the increased lipolytic rate [72]. ZAG, released by the tumour, seems to be the mediator able to activate the triacylglycerol lipase responsible for the increased lipolysis associated with cancer cachexia.

Das et al. found that genetic ablation of adipose triglyceride lipase (ATGL) in the mouse resulted in a prevention of increased lipolysis and, therefore, reduction in WAT, associated with tumour burden [73]. Interestingly, ablation of hormone-sensitive lipase (HSL) leads to similar but less marked effects [73]. Interestingly, the lipolytic ablation resulted in a preservation of skeletal muscle mass, suggesting that the breakdown of fat precedes that of skeletal muscle proteins and implicating that some signal(s) generated during the breakdown of adipocyte triacylglycerols may actually activate muscle proteolysis (Figure 2). The ablation of the mentioned lipase was also associated with a lack of activation of the main proteolytic system involved in muscle wasting during cancer, which is that of the ubiquitin-proteasome pathway [73]. In addition to the changes related to adipose tissue itself, infiltration of adipose tissue in skeletal muscle could contribute to wasting in this tissue. From this point of view Stephens et al. [74] have reported increased presence of intramyocellular lipid droplets in* rectus abdominis* muscle of cancer patients, which seems to be related to body weight loss in these patients.

### 6.2. Brown Adipose Tissue (BAT) and Energetic Inefficiency

The metabolic energetic inefficiency, linked to hypermetabolism, found in the cancer patient, seems to be responsible, together with the reduced food intake, for the negative energy balance found in the patient [1]. Hypermetabolism seems to be related with inflammation [43]. Many futile cycles are responsible for hypermetabolism, including increased Cori Cycle activity between the liver and the tumour [75], liver glycolysis/gluconeogenesis [76], muscle protein synthesis/degradation [77], and adipose tissue triacylglycerol recycling [78]. An alternative mechanism contributing to hypermetabolism is the mitochondrial uncoupling proteins, originally described in BAT. Until quite recently, BAT has been considered as a thermogenic organ in rodents. By burning fat, BAT provides fatty acids which are further oxidized but, instead of serving for mitochondrial ATP synthesis, the energy associated with the oxidative process is released as heat due to the existence in the inner mitochondrial membrane of uncoupling proteins that permeabilize? the mitochondrial membrane to the electrochemical H^+^ gradient that drives ATP synthesis [79]. In 2009, Virtanen et al. showed that BAT was present in adult humans in the upper back, in the neck, between the collarbone and shoulder, and also along the spine, suggesting also a function for BAT in humans [80]. Recent data suggest the existence of two different types of brown adipose tissue cells: in addition to the “classical” uncoupling protein 1, UCP1 positive, derived from a myf-5 cellular lineage, so-called “beige” adipose cell exists with very low UCP1 expression and is derived from a non-myf-5 cellular lineage. Beige cells have their own gene pattern expression, different from both white and brown cells, and respond preferably to irisin [81]. Until now, no information is available concerning a possible role of these cells in cancer cachexia but certainly the topic deserves future attention.

Since BAT plays a key role in thermogenesis and energy balance, it may potentially contribute to the physiologic perturbations associated with cachexia (Figure 1). Several reports in experimental animals already point out a clear activation of BAT during cancer cachexia. Recently, Tsoli et al. have demonstrated increased BAT thermogenesis in cachectic tumour-bearing mice due to increased UCP1 or lipid oxidation (CPT-1*α* and peroxisomal bifunctional enzyme (PBE), one of the four enzymes of the peroxisomal beta-oxidation pathway) [82]. The changes observed seem to be related to an activation of STAT-3, possibly via IL-6. Unfortunately, no information is available on the role of brown fat in human cancer cachexia; therefore future research on this aspect is strongly encouraged.

In addition, there is another aspect to bear into consideration related to patients with cancer. Since the observation of the existence of BAT in adult humans, a population of cells, within WAT, which has the same characteristics as brown adipocytes, have been described [83, 84]. These cells are known as BRITE (brown into white) and seem to appear in WAT under certain conditions that seem to involve the COX-2 prostaglandin pathway. Both BAT and BRITE cells are more sensitive to insulin than WAT ones; therefore they consume glucose at a higher rate. Since both insulin and IGF-1 have been reported to fuel some tumours, having more or less BAT may affect the overall systemic insulin sensitivity and thereby have an indirect influence of tumour progression.

## 7. Conclusions and Future Directions

Since human skeletal muscle represents almost 50% of body weight, research on cancer wasting has for a long time been mainly devoted to this skeletal tissue. However, cancer cachexia is indeed a multiorgan syndrome affecting many types of cells, including adipose tissues, heart, liver, gastrointestinal tract, and brain. It has been recently reported that mediators released in nonmuscle tissues, during the cachexia syndrome, may actually be directly responsible for the activation of the metabolic alterations, such as increased protein degradation [85, 86], apoptosis [87, 88], and altered regeneration [89], leading to skeletal muscle wasting.

The implications of this are important since, on the one hand, the metabolic alterations affecting all cellular types may be very relevant to the understanding of the cachexia syndrome and, secondly, the development of new therapeutic approaches may benefit from this knowledge: for instance, it may be relevant to interfering with lipolysis or with acute-phase protein synthesis to block muscle proteolysis. Therefore, future studies on this field are needed and should concentrate on unrevealing the different mediators released by nonmuscle tissues that may influence muscle metabolism and, therefore, wasting. Furthermore, another important aspect that future research should contemplate is to establish the chronological involvement of the different organs/tissues in cancer cachexia.

## References

[1] Evans W. J., Morley J. E., Argilés J. (2008). Cachexia: a new definition. *Clinical Nutrition*.

[2] Argilés J. M., López-Soriano F. J., Busquets S. (2007). Mechanisms to explain wasting of muscle and fat in cancer cachexia. *Current Opinion in Supportive and Palliative Care*.

[3] Fearon K., Strasser F., Anker S. D. (2011). Definition and classification of cancer cachexia: an international consensus. *The Lancet Oncology*.

[4] Blum D., Omlin A., Baracos V. E. (2011). Cancer cachexia: a systematic literature review of items and domains associated with involuntary weight loss in cancer. *Critical Reviews in Oncology/Hematology*.

[5] Warren S. (1932). The immediate cause of death in cancer. *The American Journal of the Medical Sciences*.

[6] Dewys W. D., Begg C., Lavin P. T. (1980). Prognostic effect of weight loss prior tochemotherapy in cancer patients. *The American Journal of Medicine*.

[7] Solheim T. S., Fayers P. M., Fladvad T. (2012). Is there a genetic cause of appetite loss?-an explorative study in 1,853 cancer patients. *Journal of Cachexia, Sarcopenia and Muscle*.

[8] Tohgo A., Kumazawa E., Akahane K., Asakawa A., Inui A. (2002). Anticancer drugs that induce cancer-associated cachectic syndromes. *Expert Review of Anticancer Therapy*.

[9] Hopkinson J. B. (2010). The emotional aspects of cancer anorexia. *Current Opinion in Supportive and Palliative Care*.

[10] Molfino A., Laviano A., Fanelli F. R. (2010). Contribution of anorexia to tissue wasting in cachexia. *Current Opinion in Supportive and Palliative Care*.

[11] Evans W. K., Makuch R., Clamon G. H. (1985). Limited impact of total parenteral nutrition on nutritional status during treatment for small cell lung cancer. *Cancer Research*.

[12] Joppa M. A., Gogas K. R., Foster A. C., Markison S. (2007). Central infusion of the melanocortin receptor antagonist agouti-related peptide (AgRP(83-132)) prevents cachexia-related symptoms induced by radiation and colon-26 tumors in mice. *Peptides*.

[13] Cheung W. W., Mak R. H. (2012). Melanocortin antagonism ameliorates muscle wasting and inflammation in chronic kidney disease. *American Journal of Physiology: Renal Physiology*.

[14] Puppa M. J., White J. P., Sato S., Cairns M., Baynes J. W., Carson J. A. (2011). Gut barrier dysfunction in the *Apc*
^*Min*/+^ mouse model of colon cancer cachexia. *Biochimica et Biophysica Acta*.

[15] Soler A. P., Miller R. D., Laughlin K. V., Carp N. Z., Klurfeld D. M., Mullin J. M. (1999). Increased tight junctional permeability is associated with the development of colon cancer. *Carcinogenesis*.

[16] Ohtani S., Terashima M., Satoh J. (2009). Expression of tight-junction-associated proteins in human gastric cancer: downregulation of claudin-4 correlates with tumor aggressiveness and survival. *Gastric Cancer*.

[17] Kufe D. W. (2009). Mucins in cancer: function, prognosis and therapy. *Nature Reviews Cancer*.

[18] Bindels L. B., Beck R., Schakman O. (2012). Restoring specific lactobacilli levels decreases inflammation and muscle atrophy markers in an acute leukemia mouse model. *PLoS ONE*.

[19] Bindels L. B., Delzenne N. M. (2013). Muscle wasting: the gut microbiota as a new therapeutic target?. *International Journal of Biochemistry and Cell Biology*.

[20] Watanabe M., Houten S. M., Mataki C. (2006). Bile acids induce energy expenditure by promoting intracellular thyroid hormone activation. *Nature*.

[21] Kojima M., Hosoda H., Date Y., Nakazato M., Matsuo H., Kangawa K. (1999). Ghrelin is a growth-hormone-releasing acylated peptide from stomach. *Nature*.

[22] Castañeda T. R., Tong J., Datta R., Culler M., Tschöp M. H. (2010). Ghrelin in the regulation of body weight and metabolism. *Frontiers in Neuroendocrinology*.

[23] Sousa-Ferreira L., Álvaro A. R., Aveleira C. (2011). Proliferative hypothalamic neurospheres express NPY, AGRP, POMC, CART and orexin-a and differentiate to functional neurons. *PLoS ONE*.

[24] Date Y., Murakami N., Toshinai K. (2002). The role of the gastric afferent vagal nerve in Ghrelin-induced feeding and growth hormone secretion in rats. *Gastroenterology*.

[25] Choi K., Roh S.-G., Hong Y.-H. (2003). The role of ghrelin and growth hormone secretagogues receptor on rat adipogenesis. *Endocrinology*.

[26] Mano-Otagiri A., Iwasaki-Sekino A., Nemoto T. (2010). Genetic suppression of ghrelin receptors activates brown adipocyte function and decreases fat storage in rats. *Regulatory Peptides*.

[27] Carreira M. C., Crujeiras A. B., Andrade S., Monteiro M. P., Casanueva F. F. (2013). Ghrelin as a GH-releasing factor. *Endocrine Development*.

[28] Wang H. S., Oh D. S., Ohning G. V., Pisegna J. R. (2007). Elevated serum ghrelin exerts an orexigenic effect that may maintain body mass index in patients with metastatic neuroendocrine tumors. *Journal of Molecular Neuroscience*.

[29] Kerem M., Ferahkose Z., Yilmaz U. T. (2008). Adipokines and ghrelin in gastric cancer cachexia. *World Journal of Gastroenterology*.

[30] Takahashi M., Terashima M., Takagane A., Oyama K., Fujiwara H., Wakabayashi G. (2009). Ghrelin and leptin levels in cachectic patients with cancer of the digestive organs. *International Journal of Clinical Oncology*.

[31] Karapanagiotou E. M., Polyzos A., Dilana K. D. (2009). Increased serum levels of ghrelin at diagnosis mediate body weight loss in non-small cell lung cancer (NSCLC) patients. *Lung Cancer*.

[32] Neary N. M., Small C. J., Wren A. M. (2004). Ghrelin increases energy intake in cancer patients with impaired appetite: acute, randomized, placebo-controlled trial. *Journal of Clinical Endocrinology and Metabolism*.

[33] Strasser F., Lutz T. A., Maeder M. T. (2008). Safety, tolerability and pharmacokinetics of intravenous ghrelin for cancer-related anorexia/cachexia: a randomised, placebo-controlled, double-blind, double-crossover study. *British Journal of Cancer*.

[34] Garcia J. M., Polvino W. J. (2007). Effect on body weight and safety of RC-1291, a novel, orally available ghrelin mimetic and growth hormone secretagogue: results of a phase I, randomized, placebo-controlled, multiple-dose study in healthy volunteers. *Oncologist*.

[35] Garcia J. M., Friend J., Allen S. (2013). Therapeutic potential of anamorelin, a novel, oral ghrelin mimetic, in patients with cancer-related cachexia: a multicenter, randomized, double-blind, crossover, pilot study. *Supportive Care in Cancer*.

[36] Lundholm K., Gunnebo L., Körner U. (2010). Effects by daily long term provision of ghrelin to unselected weight-losing cancer patients: a randomized double-blind study. *Cancer*.

[37] Adachi S., Takiguchi S., Okada K. (2010). Effects of ghrelin administration after total gastrectomy: a prospective, randomized, placebo-controlled phase II study. *Gastroenterology*.

[38] Majchrzak K., Szyszko K., Pawłowski K. M., Motyl T., Król M. (2012). A role of ghrelin in cancerogenesis. *Polish Journal of Veterinary Sciences*.

[39] Dallmann R., Weyermann P., Anklin C. (2011). The orally active melanocortin-4 receptor antagonist BL-6020/979: a promising candidate for the treatment of cancer cachexia. *Journal of Cachexia, Sarcopenia and Muscle*.

[40] Ruud J., Nilsson A., Engström Ruud L. (2013). Cancer-induced anorexia in tumor-bearing mice is dependent on cyclooxygenase-1. *Brain, Behavior, and Immunity*.

[41] Pecchi E., Dallaporta M., Jean A., Thirion S., Troadec J. D. (2008). mPGES-1 knock-out mice are resistant to cancer-induced anorexia despite the absence of central mPGES-1 up-regulation in wild-type anorexic mice. *Journal of Neuroimmunology*.

[42] Argilés J. M., Busquets S., López-Soriano F. J. (2001). Metabolic interrelationships between liver and skeletal muscle in pathological states. *Life Sciences*.

[43] Argilés J. M., Alvarez B., López-Soriano F. J. (1997). The metabolic basis of cancer cachexia. *Medicinal Research Reviews*.

[44] Andersson C., Gelin J., Iresjo B.-M., Lundholm K. (1993). Acute-phase proteins in response to tumor growth. *Journal of Surgical Research*.

[45] Fearon K. C. H., Stuart Falconer J., Slater C., McMillan D. C., Ross J. A., Preston T. (1998). Albumin synthesis rates are not decreased in hypoalbuminemic cachectic cancer patients with an ongoing acute-phase protein response. *Annals of Surgery*.

[46] Zhang L., Du J., Hu Z. (2009). IL-6 and serum amyloid A synergy mediates angiotensin II-induced muscle wasting. *Journal of the American Society of Nephrology*.

[47] Richards C. H., Roxburgh C. S. D., MacMillan M. T. (2012). The relationships between body composition and the systemic inflammatory response in patients with primary operable colorectal cancer. *PLoS ONE*.

[48] Roxburgh C. S. D., McMillan D. C. (2010). Role of systemic inflammatory response in predicting survival in patients with primary operable cancer. *Future Oncology*.

[49] Proctor M. J., Morrison D. S., Talwar D. (2011). An inflammation-based prognostic score (mGPS) predicts cancer survival independent of tumour site: a Glasgow Inflammation Outcome Study. *British Journal of Cancer*.

[50] Dumas J.-F., Goupille C., Julienne C. M. (2011). Efficiency of oxidative phosphorylation in liver mitochondria is decreased in a rat model of peritoneal carcinosis. *Journal of Hepatology*.

[51] Dumas J.-F., Peyta L., Couet C., Servais S. (2013). Implication of liver cardiolipins in mitochondrial energy metabolism disorder in cancer cachexia. *Biochimie*.

[52] Martignoni M. E., Dimitriu C., Bachmann J. (2009). Liver macrophages contribute to pancreatic cancer-related cachexia. *Oncology Reports*.

[53] Olivan M., Springer J., Busquets S. (2012). Theophylline is able to partially revert cachexia in tumour-bearing rats. *Nutrition and Metabolism*.

[54] Der-Torossian H., Gourin C. G., Couch M. E. (2012). Translational implications of novel findings in cancer cachexia: the use of metabolomics and the potential of cardiac malfunction. *Current Opinion in Supportive and Palliative Care*.

[55] Schünemann M., Anker S. D., Rauchhaus M. (2008). Cancer fatigue syndrome reflects clinically non-overt heart failure: An approach towards oncocardiology. *Nature Clinical Practice Oncology*.

[56] Tian M., Nishijima Y., Asp M. L., Stout M. B., Reiser P. J., Belury M. A. (2010). Cardiac alterations in cancer-induced cachexia in mice. *International Journal of Oncology*.

[57] Mühlfeld C., Das S. K., Heinzel F. R. (2011). Cancer induces cardiomyocyte remodeling and hypoinnervation in the left ventricle of the mouse heart. *PLoS ONE*.

[58] Tian M., Asp M. L., Nishijima Y., Belury M. A. (2011). Evidence for cardiac atrophic remodeling in cancer-induced cachexia in mice. *International Journal of Oncology*.

[59] Cosper P. F., Leinwand L. A. (2011). Cancer causes cardiac atrophy and autophagy in a sexually dimorphic manner. *Cancer Research*.

[60] Wysong A., Couch M., Shadfar S. (2011). NF-*κ*B inhibition protects against tumor-induced cardiac atrophy in vivo. *American Journal of Pathology*.

[61] Zhou X., Wang J. L., Lu J. (2010). Reversal of cancer cachexia and muscle wasting by ActRIIB antagonism leads to prolonged survival. *Cell*.

[62] Drott C., Lundholm K. (1990). Glucose uptake and amino acid metabolism in perfused hearts from tumor-bearing rats. *Journal of Surgical Research*.

[63] Hyltander A., Drott C., Korner U., Sandstrom R., Lundholm K. (1991). Elevated energy expenditure in cancer patients with solid tumours. *European Journal of Cancer*.

[64] Argilés J. M., López-Soriano J., Almendro V., Busquets S., López-Soriano F. J. (2005). Cross-talk between skeletal muscle and adipose tissue: a link with obesity?. *Medicinal Research Reviews*.

[65] Argilés J. M., López-Soriano F. J., Busquets S. (2009). Therapeutic potential of interleukin-15: a myokine involved in muscle wasting and adiposity. *Drug Discovery Today*.

[66] Moreno-Navarrete J. M., Ortega F., Serrano M. (2013). Irisin is expressed and produced by human muscle and adipose tissue in association with obesity and insulin resistance. *The Journal of Clinical Endocrinology & Metabolism*.

[67] Boström P., Wu J., Jedrychowski M. P. (2012). A PGC1-*α*-dependent myokine that drives brown-fat-like development of white fat and thermogenesis. *Nature*.

[68] Shan T., Liang X., Bi P., Kuang S. (2013). Myostatin knockout drives browning of white adipose tissue through activating the AMPK-PGC1-Fndc5 pathway in muscle. *FASEB Journal*.

[69] Dahlman I., Mejhert N., Linder K. (2010). Adipose tissue pathways involved in weight loss of cancer cachexia. *British Journal of Cancer*.

[70] Batista J., Neves R. X., Peres S. B. (2012). Heterogeneous time-dependent response of adipose tissue during the development of cancer cachexia. *Journal of Endocrinology*.

[71] Batista M. L., Peres S. B., McDonald M. E. (2012). Adipose tissue inflammation and cancer cachexia: possible role of nuclear transcription factors. *Cytokine*.

[72] Mracek T., Stephens N. A., Gao D. (2011). Enhanced ZAG production by subcutaneous adipose tissue is linked to weight loss in gastrointestinal cancer patients. *British Journal of Cancer*.

[73] Das S. K., Eder S., Schauer S. (2011). Adipose triglyceride lipase contributes to cancer-associated cachexia. *Science*.

[74] Stephens N. A., Skipworth R. J. E., MacDonald A. J., Greig C. A., Ross J. A., Fearon K. C. H. (2011). Intramyocellular lipid droplets increase with progression of cachexia in cancer patients. *Journal of Cachexia, Sarcopenia and Muscle*.

[75] Zentella A., Manogue K., Cerami A. (1993). Cachectin/TNF-mediated lactate production in cultured myocytes is linked to activation of a futile substrate cycle. *Cytokine*.

[76] Llovera M., López-Soriano F. J., Argilés J. M. (1993). Effects of tumor necrosis factor-*α* on muscle-protein turnover in female Wistar rats. *Journal of the National Cancer Institute*.

[77] Edén E., Edström S., Bennegård K., Scherstén T., Lundholm K. (1984). Glucose flux in relation to energy expenditure in malnourished patients with and without cancer during periods of fasting and feeding. *Cancer Research*.

[78] Beck S. A., Tisdale M. J. (2004). Effect of cancer cachexia on triacylglycerol/fatty acid substrate cycling in white adipose tissue. *Lipids*.

[79] Argilés J. M., Busquets S., López-Soriano F. J. (2002). The role of uncoupling proteins in pathophysiological states. *Biochemical and Biophysical Research Communications*.

[80] Virtanen K. A., Lidell M. E., Orava J. (2009). Functional brown adipose tissue in healthy adults. *The New England Journal of Medicine*.

[81] Wu J., Boström P., Sparks L. M. (2012). Beige adipocytes are a distinct type of thermogenic fat cell in mouse and human. *Cell*.

[82] Tsoli M., Moore M., Burg D. (2012). Activation of thermogenesis in brown adipose tissue and dysregulated lipid metabolism associated with cancer cachexia in mice. *Cancer Research*.

[83] Pisani D. F., Djedaini M., Beranger G. E. (2011). Differentiation of human adipose-derived stem cells into ‘brite’ (brown-in-white) adipocytes. *Frontiers in Endocrinology*.

[84] Sharp L. Z., Shinoda K., Ohno H. (2012). Human BAT possesses molecular signatures that resemble beige/brite cells. *PLoS ONE*.

[85] Llovera M., Garcia-Martinez C., Agell N., Lopez-Soriano F. J., Argiles J. M. (1995). Muscle wasting associated with cancer cachexia is linked to an important activation of the ATP-dependent ubiquitin-mediated proteolysis. *International Journal of Cancer*.

[86] Bossola M., Muscaritoli M., Costelli P. (2001). Increased muscle ubiquitin mrna levels in gastric cancer patients. *American Journal of Physiology: Regulatory Integrative and Comparative Physiology*.

[87] van Royen M., Carbó N., Busquets S. (2000). DNA fragmentation occurs in skeletal muscle during tumor growth: a link with cancer cachexia?. *Biochemical and Biophysical Research Communications*.

[88] Mehl K. A., Davis J. M., Berger F. G., Carson J. A. (2005). Myofiber degeneration/regeneration is induced in the cachectic *Apc*
^*Min*/+^ mouse. *Journal of Applied Physiology*.

[89] Ametller E., Busquets S., Fuster G. (2011). Formoterol may activate rat muscle regeneration during cancer cachexia. *Insciences Journal*.

